# Moving towards health promoting schools: effectiveness of an educational intervention to improve knowledge, attitude and beliefs regarding heart attack, and CPR knowledge in high school students in Lebanon

**DOI:** 10.3389/fpubh.2024.1355766

**Published:** 2024-05-30

**Authors:** Mohamad Abdelkhalik, Eliannore Boutros, Khalid Trad, Oulyana Arafat, Mohamad Nihad Alyousfi, Carmel Bouclaous

**Affiliations:** Gilbert and Rose-Marie Chagoury School of Medicine, Lebanese American University, Byblos, Lebanon

**Keywords:** heart attack and cardiopulmonary resuscitation, students, Lebanon, health promoting schools, health literacy, knowledge, attitude, beliefs

## Abstract

**Background:**

Health promoting schools (HPS) prioritize the health of students and community. One important target of HPS is noncommunicable diseases (NCDs), including prevention of heart attacks, due to their burden on healthcare.

**Objective:**

This study assesses the effectiveness of an educational intervention to promote knowledge of signs and symptoms, beliefs and attitudes towards heart attack, and promote knowledge of Cardiopulmonary resuscitation (CPR).

**Methods:**

The intervention consisted of a 6-minute educational video between a pre-and post-survey. Among other questions, the survey included the Calgary Charter on Health literacy scale, the acute coronary syndrome response index questionnaire, and items assessing knowledge of CPR.

**Results:**

A total of 401 high school students participated (58.9% females). Few students had adequate baseline knowledge of heart attack symptoms (22%) and CPR (7%). The sample showed moderate level of health literacy (12 ± 2.7). Chest pain was the most identified symptom (95%) while abdominal pain was the least identified (14.25%). The intervention significantly increased knowledge, beliefs and attitudes towards heart attack, and knowledge of CPR (*p* < 0.001). Following the intervention, 83.2% of students demonstrated sufficient knowledge of heart attack symptoms, and 45% exhibited adequate knowledge of CPR. Variables predictive of better attitude, in other words higher confidence in recognizing and reacting to symptoms of heart attack, included having higher health literacy and prior knowledge of risk factors (*p* < 0.05). Needing help reading medical instructions sometimes predicted worse belief in their capacity to act if they experienced or witnessed a heart attack [score (*p* < 0.05)]. It was also predictive of worse attitude towards heart attack (OR = 0.18).

**Conclusion:**

High school students in Lebanon lack appropriate knowledge, attitudes, and beliefs toward heart attack, and lack CPR qualifications. Scale up of this educational initiative, along with training of teachers and school personnel, can be used as part of a holistic HPS program aimed at raising awareness of heart attack and first responder preparedness.

## 1 Introduction

The Health Promoting Schools (HPS) approach introduced in the late 1980s, embeds health into the school mission, and encourages the adoption of healthy decisions and behaviors in school settings (1). HPS are educational institutions that prioritize the health of students, faculty, staff, and the wider community, and strive for safer and healthier school environments.

One key feature of HPS approach is flexibility, allowing schools to focus on the most pressing topics in their community, while ensuring a timely and cost-effective implementation of global objectives (2). For example, at the height of the COVID-19 pandemic, schools played an important role in educating students and their families, about hygiene, social distancing, and the identification of alarm symptoms requiring medical care (3). Similarly, HPS programs and schemes related to body mass index, physical activity, and nutritional habits, were impactful in changing habits (4). The effectiveness of health promotion approaches in schools highly depends on the close collaboration between medical professionals and other stakeholders who issue HPS curricula, and education professionals who deliver the message, to ensure understanding of the material and objectives (5). The presence of qualified teachers, supportive school administration, sustainable funding, and long-term planning contribute to the success rates of HPS approaches (6, 7).

HPS seem paramount for addressing the United Nation’s Sustainable Development Goals mainly SDGs 3 and 4 related to good health and wellbeing and quality education respectively, as well as the goals of the World Health Organization (WHO) global action plan for non-communicable diseases (NCDs) prevention, 2013–2020 (8).

NCDs are evolving into a growing burden worldwide. In Lebanon, cardiovascular diseases (CVDs) account for 47% of all deaths (9). This is attributed to a worsening cardiovascular profile in the population with high risk factors like obesity (41.5%), alcohol (18.96%), and smoking (43.1%) (10). The Lebanese National Non-Communicable Disease Prevention and Control Plan 2016–2020 (NCD-PCP) was established by the Ministry of Public Health (11), and was based on a multisectoral approach to health promotion and community-based primary prevention of NCDs. Nonetheless, the program lacked an assessment of baseline knowledge, beliefs, and attitudes of the general population concerning NCDs, notably CVDs, and first aid care of cardiovascular emergencies. It also failed to involve school-based initiatives to increase students’ awareness of CVDs, although school-based health promotion has been shown to significantly impact long-term health outcomes and risk-behaviors (12).

Studies have also documented a gap in knowledge of CVDs, heart attacks and their symptoms, and a need for emergency medical care in the adult population in Lebanon and the wider Middle East (13–19). This lack of recognition of heart attack signs and symptoms by the general population is concerning, as it causes delays in seeking medical attention and treatment, leading to poorer prognosis and increased mortality. This delay has been attributed to a lack of recognition of the signs and symptoms of heart attack and hesitancy to perform cardiopulmonary resuscitation (CPR), which has been shown to increase chances of survival by 2–3 folds (20). This hesitancy has been linked to lack of training and lack of prior experience in performing CPR (21).

Several educational campaigns were initiated globally to increase knowledge of signs and symptoms of heart attack and reduce the delay in hospital arrival. A face-to-face session and counseling on common symptoms of heart attack and appropriate response to such an event increased knowledge in adults who were diagnosed with coronary artery disease (CAD) in the previous 6 months (22), and was maintained after 12 months. The use of a personal resuscitation manikin with video education enhanced basic life support (BLS) performance of hospital staff with limited or no direct patient contact, for up to six months in Norway (23). Moreover, students have demonstrated a stronger capacity to learn, receive, and retain new information related to CPR compared to older individuals (24–26). Students aged 15 to 16 years, who took CPR instruction were not only able to initiate BLS in times of need, but also created an information cascade by sharing their knowledge with their families (27).

Accordingly, the main objectives of this research are to (1) assess baseline knowledge, beliefs, and attitudes of high school students regarding heart attack signs and symptoms, (2) assess baseline knowledge of CPR among high school students, (3) implement an educational intervention based on a self-developed video, and (4) assess the change in baseline knowledge, beliefs and attitudes after the educational session.

## 2 Methods

### 2.1 Study design

This interventional study, with a pre-post design, consisted of a pretest, an educational video, and a posttest. The study was conducted between March and June 2023 following approval of the Lebanese American University’s Institutional Review Board. The written consent of school directors and parents was secured prior to each educational session. Participants were also required to provide written consent before partaking in the study and after reviewing its objectives. Participation was voluntary, and participants were allowed to opt out any time without repercussions. The data collected was strictly confidential with the use of a unique identification code per participant (created by the participant based on the first two letters of their name and their father’s name along with the day of birth). The unique identification code was used in the posttest questionnaire to allow the research team to link the pre and post submissions.

### 2.2 Instruments

The research team created a 6-minute educational video based on AHA guidelines, with assistance from the Center for Innovative Learning and a nurse from the Clinical Simulation Center at the Lebanese American University. The video covered heart attack epidemiology, risk factors, signs and symptoms, and performance of hands-only CPR. It was validated by a cardiologist prior to its use in the study. The research team ensured that it covered all topics asked about in the pretest.

The pretest was composed of seven different sections. The first section aimed to collect the demographic characteristics of the participants as well as prior experience with or knowledge of heart attacks. Section 2 evaluated health literacy (HL) level based on the Calgary Charter on Health Literacy scale. It assessed how difficult it is for students to look for health information, understand, evaluate, communicate, and make decisions based on this information (28). The third section targeted the risk factors for heart attack using nine questions (29). Sections 4 addressed knowledge of the signs and symptoms of heart attack (30). The knowledge questions were scored as a percentage, with ≥70% correct response as cutoff for sufficient knowledge. Sections 5 and 6 covered attitude and belief toward heart attack, and were recorded on a 4-point Likert scale, and summative scores were calculated (30). The five attitude elements evaluated one’s confidence in identifying symptoms of Acute Coronary Syndrome (ACS) and seeking assistance (score range: 5 to 20). There are seven belief items probing one’s intended actions if they were to experience or witness a heart attack (score range: 7 to 28). The Cronbach’s alpha coefficient for the validated Arabic version was 0.78 for signs and symptoms, 0.80 for attitudes, and 0.52 for beliefs (17). The last section consisted of eight questions covering CPR knowledge (31). Each correct answer is given a score of 1, and ≥ 60% correct responses indicate CPR qualification. The questions were translated to Arabic and back-translated, and discrepancies were rectified.

The posttest only included the items from the pretest that asked about heart attack risk factors, signs, and symptoms, attitudes, and beliefs, as well as CPR knowledge.

### 2.3 Sample and setting

Participants were Arabic-speaking high school students, 16–19 years of age, in grades 11 and 12 of the following schools: Majdal Anjar Official Secondary School, Le Lycee National, Learner’s World International Schools – Universal School of Lebanon (LWIS-USL), and Daughters of Charity School.

### 2.4 Data collection

The research team visited the schools. Groups of 15–30 students at a time participated in the study. Students were asked to fill the online pretest, watch the video on a common screen in class, and fill an online posttest questionnaire. Students were allowed by the school administration to bring their personal phones or laptops to school for use on the day of the intervention.

### 2.5 Statistical analysis

The data was analyzed using SPSS 28 and STATA 16. Categorical variables were presented as frequencies and percentages, while continuous variables were summarized using means and standard deviations. A paired t-test was employed to compare pretest and posttest means. Exploration of the relationship between knowledge, attitudes, beliefs, and CPR knowledge with other predictors was conducted. Predictors included age, sex, governorate, nationality, socioeconomic status, help needed to read medical instructions, health literacy level, medical and social history of heart attack, and heart attack risk factor (9 factors). For categorical dependent variables, and for continuous dependent variables, both simple and multiple logistic regression analyses were conducted.

The desired sample size was 384 participants as per the following formula (32), assuming a confidence level of 95%, margin of error of 5%, and population proportion of 50%:


$$n=\frac{z^{2}\times p\;\left(1-p\right)}{\varepsilon^{2}}$$

## 3 Results

### 3.1 Sample characteristics

Table 1 presents the sample characteristics. Participants (*N* = 401) had a mean age of 16.9 years ±1.92 with a majority of females (58.9%), and mainly Lebanese (95.8%). The mean HL score was 12 ± 2.7, indicating a moderately sufficient level.

**Table 1:** General characteristics of the sample (*N* = 401).

Variable	Number	Percentage
Gender		
Female	236	58.9
Male	165	41.1
Governorate		
Mount Lebanon	110	27.4
Bekaa	39	9.7
Baalbak	1	0.2
North	130	32.4
Akkar	2	0.5
South	4	1
Beirut	115	28.7
Nationality		
Lebanese	384	95.8
Syrian	10	2.5
Palestinian	7	1.7
Seeking assistance with medical instructions		
Never	79	19.7
Rarely	112	27.9
Sometimes	150	37.4
Mostly	36	9
Always	24	6
Health literacy (mean ± SD)		
Score may range from 5 to 20	401	12.0 (±2.7)

### 3.2 Knowledge, attitudes, and beliefs about heart attack before and after the educational intervention

Table 2 displays the pretest and posttest scores on the knowledge, attitude, and beliefs on heart attack, and CPR knowledge of the participants. Prior to the intervention, participants exhibited a 12.2 ± 3.2 mean score on knowledge of signs and symptoms, with 22% achieving a score higher than 70%. This indicates very low level of knowledge.

**Table 2:** Change in knowledge, attitudes, beliefs toward signs and symptoms of heart attack as well as cardiopulmonary resuscitation knowledge after viewing the educational video.

Score	Test	Mean (±SD)	Mean difference between post and pretests (±SD)	95% CI	*p*-value
Knowledge score (possible range: 0 to 21)	Pretest	12.25 (3.2)	5.52 (4)	(5.1, 5.9)	**<0.001**
	Posttest	17.78 (3.3)			
Attitudes score (possible range: 5 to 20)	Pretest	9.76 (2.8)	3.60 (3.2)	(3.3, 3.9)	**<0.001**
	Posttest	13.39 (3.1)			
Beliefs score (possible range: 7 to 28)	Pretest	17.68 (2.6)	−0.48 (3)	(−0.78, −0.18)	**0.001**
	Posttest	17.19 (2.9)			
Cardiopulmonary resuscitation knowledge (possible range: 0 to 8)	Pretest	2.44 (1.4)	1.74 (1.9)	(1.5, 1.9)	**<0.001**
	Posttest	4.19 (1.7)			

In bold, significant difference at *p* < 0.05.

Following the intervention, there was a significant improvement in mean knowledge score (*p* < 0.001) by 1.5 times with 83.2% of participants achieving a score above 70%. Similarly, attitudes and beliefs scores were significantly improved (*p* < 0.001).

### 3.3 Identification of signs and symptoms of heart attack before and after the educational intervention

Table 3 displays participants’ recognition of each sign and symptom of heart attack. Before the intervention, the most identified symptom was chest pain (95%), while lower abdominal pain, back pain, cough, heartburn, jaw pain, nausea, vomiting, and neck pain were identified by less than 50% of participants. Post intervention, chest pain remained the most recognized symptom (98%). Heartburn was least identified (63.1%), but with no sign or symptom scoring less than 50%. Thus, the intervention significantly improved knowledge of all signs and symptoms of heart attack.

**Table 3:** Frequency and percentage of recognition, by 401 adolescents of the signs and symptoms of heart attack before and after educational video.

Signs and symptoms	Pretest		Posttest	
	Frequency	Percentage	Frequency	Percentage
Lower abdominal pain	57	14.2	319	79.55
Arm pain or shoulder pain	208	51.9	348	86.8
Arm paralysis	245	61.1	349	87
Back pain	89	22.2	296	73.8
Chest pain/pressure/tightness	381	95	393	98
Chest discomfort/heaviness/burning/tenderness	340	84.8	357	89
Cough	130	32.4	266	66.3
Dizziness/lightheadedness	270	67.3	391	97.5
Headache	217	54.1	333	83
Heartburn/indigestion/stomach problems	100	24.9	253	63.1
Jaw pain	84	20.9	266	66.33
Loss of consciousness/fainting	331	82.5	388	96.75
Nausea/vomiting	152	37.9	336	83.8
Neck pain	121	30.2	314	78.3
Numbness/tingling in arm or hand	269	67.1	381	95
Pale/ashen/loss or change of color	320	79.8	315	78.55
Palpitations/rapid heart rate	357	89	352	87.8
Shortness of breath/difficulty breathing	368	91.8	392	97.75
Slurred speech	262	65.3	328	81.8
Sweating	290	72.3	384	95.8
Weakness/fatigue	322	80.3	368	91.8
Participants with > 70% correct answers	88	**22**	334	**83.2**

The explanation is provided beneath the table (*p* is significant).

### 3.4 CPR knowledge before and after the educational intervention

Baseline knowledge of CPR was 2.44 ± 1.74, with only 7% having more than 60% correct answers. The question “Do you know the correct steps for cardiopulmonary resuscitation?” yielded the highest proportion (47%) of accurate responses. Most participants (78%) did not know the appropriate site for chest compression. Overall, knowledge, of CPR was very low. After watching the video, there was a significant increase in mean score for CPR (*p* < 0.001), reaching 4.19 (almost doubling). As many as 45% of participants achieved a score higher than 60%. The question “Do you know the frequency of chest compressions for adults?” achieved the highest percentage of correct answers (70%).

### 3.5 Association with health literacy (HL) post-intervention

Better attitude towards heart attack indicates higher confidence in recognizing heart attack symptoms and seeking help. The odds of having better attitude was 1.22 times higher among participants with higher HL level, and 2.57 times higher in participants who were familiar with heart attack risk factors. No significant association was found between HL and knowledge or beliefs of heart attacks. On the other hand, always needing help to read medical instructions was predictive of worse attitude towards heart attack (OR = 0.18).

A higher score on beliefs indicates better expectations and actions when encountering a heart attack incident. In our study, prior knowledge about heart attack was a significant predictor of more positive beliefs (OR = 6.28) whereas needing help to read medical instructions sometimes (OR = 0.26) reflected negative beliefs.

## 4 Discussion

CVDs are the leading cause of mortality in Lebanon, accounting for around half of all annual fatalities (9). Despite national efforts to address CVDs, they remain prominent notably due to worsening cardiovascular profile of the population, and difficulties in establishing and sustaining related health-promoting and preventative practices. Thus, it seems necessary to educate the public, and particularly students, about early recognition of heart attacks and performance of bystander CPR until emergency services arrive. By focusing on high school students, our study recognizes the essential role that teenagers play in sharing health information with their families and communities, possibly increasing the intervention’s impact. One effective approach for early action on disease risk factors is to create a supportive school environment and promote positive health behaviors through HPS. However, a study involving 50 Lebanese schools showed that, although the majority had health-related initiatives, less than half conducted a program evaluation or set a plan for improvement of their health promotion strategy (33).

Our results on HL add to existing literature that shows that a majority of Lebanese adolescents (73.2%) have moderate level of HL (34). In fact, higher HL appears to be a predictor of positive attitude towards heart attack among Lebanese students. Hence, the development of HPS and the implementation of HL related to CVDs may reduce heart attack risk factors and promote early recognition and call for help. Moreover, moderate to high levels of HL in Lebanese adolescents was shown to significantly lower their odds of adopting risky health behaviors like smoking, consuming alcohol, adopting a sedentary lifestyle, and being overweight (34). Similarly, exposure to sexuality education in schools increased the likelihood of Lebanese adolescents adopting protective behavior on their first sexual encounter; this is particularly noteworthy, as schoolteachers were reported to be the main source of information on sexual and reproductive health (35).

Our study showed that Lebanese high school students had poor knowledge of heart attack signs and symptoms. Additionally, participants identified the typical symptoms of a heart attack (like chest pain) more readily than atypical symptoms. This was consistent with previous studies from Lebanon and Saudi Arabia (17, 19). Other studies in Jordan, Kuwait, and Saudi Arabia showed that difficulty breathing was the most known symptom (18, 36, 37). In our study, abdominal pain was the least identified symptom. In contrast, headache was the least identified symptom in Italy (3.5%) (38), and pain or discomfort in the arm or shoulder was least identified in South Korea (53.8%) (39). Nevertheless, a systematic review suggests that a typical characterization of heart attack is impossible, especially among patients with comorbidities (40). Thus, given the high incidence of atypical presentations of acute heart attack, it is necessary to educate students about all possible prodromal symptoms, and not only the common clinical picture of chest pain and dyspnea.

Our study demonstrated poor baseline knowledge of CPR. A similar level of knowledge was found in students in Northern Ireland, where only 4.3% achieved a knowledge score of more than 70% (41). One of the main reasons for the observed lack in CPR knowledge was inadequate or absent training, which in turn was linked to lack of time or interest, lack of needed infrastructure, and not knowing the timing and location of available CPR training sessions (42, 43). Nevertheless, despite their inadequate knowledge, students had positive attitudes towards learning CPR (44, 45). Those findings show that a positive attitude towards learning and performing CPR along with the perception of self-efficacy are essential determinants of people’s willingness to perform bystander BLS prior to arrival of professional help (46, 47). Ensuring this readiness fulfills the 5th and 6th standards of the WHO Global Standards and Indicators for HPS (48), whereby the school expands its health-promoting resources to include teaching and non-teaching staff as well as parents (standard 5), and initiates new partnerships to contribute to the quality, sustainability, and impact of health promotion initiatives (standard 6). However, knowledge alone is not enough. It is important to instil the understanding that CPR, even if done poorly, is better than no CPR.

The students who always required assistance in reading medical instructions had less confidence in recognizing a heart attack incidence and adopted a rather negative attitude. Reading and understanding basic health information constitutes functional HL. It has been reported that around 66% of the Lebanese population have insufficient functional HL, associated with low education, low socioeconomic status, and being a widow (49). Another local study associated higher HL levels and sufficient numeracy skills to healthy behaviors like exercise, healthy diet, regular glucose monitoring, and comprehension of information related to diabetes (50). Given that a vast majority of Lebanese adolescents spend extended periods online, exceeding two hours daily (34), it is essential to promote their skills to assess the accuracy and reliability of information, and provide trustworthy sources for health education (51).

Our findings were consistent with existing literature showing that prior knowledge of heart attacks, from reading information about heart attack symptoms in the media or being taught about ACS symptoms by a healthcare professional, predicted better belief in one’s ability to respond to a heart attack occurring in front of them (52). Additionally, consistent with the literature, requiring assistance in understanding medical instructions was predictive of worse belief toward heart attack.

Our study showed that a brief educational video based on the AHA guidelines and tailored to high school students can improve their knowledge, attitudes, and beliefs towards heart attack as well as CPR knowledge. Video-based interventions have been shown to be effective in improving knowledge about heart attack among adult patients aged 40 to 60 years in the United States (53), patients’ with history of heart attack in Australia (22), hospital employees in Norway (23), college students in Japan (54), family members of hospitalized heart attack patients in the United States and China (55, 56), and school students aged 7 to 12 years in Portugal (25). However, there was no difference in overall mortality, total revascularizations, or hospitalizations between participants who got education, through video-based intervention or through healthcare professionals, as part of cardiac rehabilitation, and people in control groups (57).

Our video was balanced in terms of cognitive load, visual representations, and student engagement. The script was presented in Arabic to avoid language barrier. In addition, our intervention was delivered in small groups of 15–30 participants to ensure adequate information acquisition. This was developed in response to prior research that showed that large classes were a reason for failure of HPS initiatives (6, 7, 58). In larger classes, instructors struggle to offer attention to each student, there is less interaction between instructors and students, and teachers and school personnel struggle to monitor the health and well-being of each student. School administrators welcomed our project and instructors expressed a strong interest in learning more about heart attacks and CPR. Our initiative is worth scaling up because it provides a cost-effective means to increase Lebanese students’ health-related knowledge and their personal agency. This is particularly important in view of the surge of symptoms of anxiety and mental strain among Lebanese adolescents and young adults following the corona pandemic, and other major stressors like the political instability, the economic collapse, and the Beirut port explosion (34, 59, 60).

### 4.1 Recommendations

Given the high prevalence of CVDs and related mortalities in Lebanon, along with the poor awareness of risk factors, signs, symptoms, and CPR performance, it seems necessary to educate students on the first aid requirement of heart attack. Developing a plan for Lebanon, according to the WHO HPS implementation global standards in schools (48) could address the heart attack risk factor profile of the population by implementing physical education programs, encouraging good eating habits by providing healthy food alternatives in school cafeterias, implementing nutrition education programs, and engaging kids in activities linked to growing food, cooking, and appreciating healthful meals, establishing counseling services in schools, and training educators to recognize indicators of mental health difficulties in a supportive and non-judgmental atmosphere.

This plan would require attention to legislation involving: (1) the development and implementation of national policies that support health promotion in schools (2) the collaboration with government bodies, education authorities, and health organizations to integrate health promotion into the educational system (3) and the integration of age-appropriate and culturally sensitive health education into the curriculum.

This strategy would preferably account for students’ baseline knowledge, attitudes, and beliefs towards CVDs, their individual health literacy level, and the perception of their role and capabilities in emergency situations.

### 4.2 Limitations

Our study design did not capture sustained changes in knowledge, attitudes, and behaviors because it focused on immediate pre-post comparisons. Despite the researchers’ assurance that responses would remain anonymous, a few students exhibited Hawthorne effect in that they had increased awareness of being surveyed and may have shown exaggerated positive responses or reliance on peers for common knowledge questions such as the emergency contact number for the Lebanese Red Cross. Our study lacked a control group, which makes it difficult to establish a causal relation between the educational video and the observed improvement in knowledge, attitudes, and beliefs.

## 5 Conclusion

This study showed that high school students in Lebanon had insufficient baseline knowledge of heart attack symptoms and CPR. An intervention, based on an educational video, proved to be efficient, time-saving, and practical. This can be used as part of a health promoting schools program aimed at raising awareness of heart attack and first responder preparedness. The intervention may be extended to teachers and other school personnel.

## Data availability statement

The raw data supporting the conclusions of this article will be made available by the authors, without undue reservation.

## Ethics statement

The study involving humans were approved by the Lebanese American University’s Institutional Review Board. The study were conducted in accordance with the local legislation and institutional requirements. Written informed consent for participation in this study was provided by the participants’ legal guardians/next of kin.

## Author contributions

MoA: Conceptualization, Data curation, Investigation, Methodology, Writing – original draft, Writing – review & editing. EB: Conceptualization, Investigation, Methodology, Writing – original draft. KT: Conceptualization, Data curation, Investigation, Methodology, Writing – original draft. OA: Conceptualization, Investigation, Methodology, Writing – original draft. MNA: Formal analysis, Validation, Writing – original draft, Data curation. CB: Conceptualization, Data curation, Formal analysis, Investigation, Methodology, Project administration, Resources, Supervision, Validation, Visualization, Writing – original draft, Writing – review & editing.

## References

[1] World Health Organization: WHO [Internet]. (2021). Available at: https://www.who.int/health-topics/health-promoting-schools#tab=tab_1.

[2] Meiklejohn S Peeters A Palermo C. Championing health promoting schools: a secondary school case study from Victoria, Australia. Health Educ J. (2020) 80:187–98. doi: 10.1177/0017896920961121

[3] Sormunen M Lattke L Leksy K Dadaczynski K Sakellari E Velasco V. Health promoting schools and COVID-19: preparing for the future. Scand J Public Health. (2022) 50:655–9. doi: 10.1177/14034948221091155, PMID: 35491938 PMC9361414

[4] Liu CH Chang FC Niu YZ Liao LL Chang YJ Liao Y. Students’ perceptions of school sugar-free, food and exercise environments enhance healthy eating and physical activity. Public Health Nutr. (2021) 25:1762–70. doi: 10.1017/S1368980021004961 PMC9991674 34933694

[5] Moynihan S Jourdan D McNamara M. An examination of health promoting schools in Ireland. Health Educ. (2016) 116:16–33. doi: 10.1108/HE-03-2014-0045

[6] Leger L Young I Blanchard C Perry M. Promoting health in schools: From evidence to action [internet]. Paris: IUHPE (2008) Available at: http://www.iuhpe.org/images/PUBLICATIONS/.

[7] Turunen H Sormunen M Jourdan D Seelen J Buijs G. Health promoting schools—a complex approach and a major means to health improvement. Health Promot Int. (2017) 32:177–84. doi: 10.1093/heapro/dax001, PMID: 28384373

[8] Dadaczynski K Jensen BB Viig NG Sormunen M von Seelen J Kuchma V. Health, well-being and education. Health Educ. (2020) 120:11–9. doi: 10.1108/HE-12-2019-0058

[9] W.H.O. Noncommunicable diseases country profiles 2018, World Health Organization [Internet] (2018). Available at: https://www.who.int/publications/i/item/9789241514620.

[10] Ismaeel HA Almedawar MM Breidy J Nasrallah M Nakhoul N Mouneimne Y. Worsening of the cardiovascular profile in a developing country: the greater Beirut area cardiovascular cohort. glob. Heart. (2018) 13:275–83. doi: 10.1016/j.gheart.2018.03.001 29716848

[11] Adib S Rady A. Non communicable disease prevention and control plan (NCD-PCP) for Lebanon 2015–2020. Lebanon: Ministry of Public Health (2014).

[12] Bay J Hipkins R Siddiqi K Huque R Dixon R Shirley D. School-based primary NCD risk reduction: education and public health perspectives. Health Promot Int. (2016) 32:daw096. doi: 10.1093/heapro/daw096 28011654

[13] Al-Safi SA Alkofahi AS El-Eid HS. Public response to chest pain in Jordan. Eur J Cardiovasc Nurs. (2005) 4:139–44. doi: 10.1016/j.ejcnurse.2005.03.001, PMID: 15904884

[14] Memis S Evci ED Ergin F Beser E. A population-based study on awareness of heart attack in Aydin city-Turkey. Anadolu Kardiyol Derg. (2009) 9:304–10. 19666433

[15] Noureddine S Froelicher ES Sibai AM Dakik H. Response to a cardiac event in relation to cardiac knowledge and risk perception in a Lebanese sample: a cross sectional survey. Int J Nurs Stud. (2010) 47:332–41. doi: 10.1016/j.ijnurstu.2009.07.002, PMID: 19674744

[16] Noureddine S Massouh A Froelicher ES. Perceptions of heart disease in community-dwelling Lebanese. Eur J Cardiovasc Nurs. (2013) 12:56–63. doi: 10.1177/1474515111430899, PMID: 22414582

[17] Noureddine S Dumit NY Maatouk H. Patients’ knowledge and attitudes about myocardial infarction. Nurs Health Sci. (2020) 22:49–56. doi: 10.1111/nhs.12642 31411818

[18] Mujamammi AH Alluhaymid YM Alshibani MG Alotaibi FY Alzahrani KM Alotaibi AB. Awareness of cardiovascular disease associated risk factors among Saudis in Riyadh City. J Fam Med Prim Care. (2020) 9:3100–5. doi: 10.4103/jfmpc.jfmpc_458_20, PMID: 32984180 PMC7491763

[19] Al Harbi KM Alluhidan WA Almatroudi MI Almuhanna NI Alotaibi NM. Knowledge and attitude of general people towards symptoms of heart attack and the impact of delay time in Riyadh, Saudi Arabia. Cureus. (2022) 14:32758. doi: 10.7759/cureus.32758 PMC9767791 36561329

[20] Handley AJ Koster R Monsieurs K Perkins GD Davies S Bossaert L. European resuscitation council guidelines for resuscitation 2005. Section 2. Adult basic life support and use of automated external defibrillators. Resuscitation. (2005) 67:S7–S23. doi: 10.1016/j.resuscitation.2005.10.007 16321717

[21] Kuramoto N Morimoto T Kubota Y Maeda Y Seki S Takada K. Public perception of and willingness to perform bystander CPR in Japan. Resuscitation. (2008) 79:475–81. doi: 10.1016/j.resuscitation.2008.07.005, PMID: 18805615

[22] Buckley T McKinley S Gallagher R Dracup K Moser DK Aitken LM. The effect of education and counselling on knowledge, attitudes and beliefs about responses to acute myocardial infarction symptoms. Eur J Cardiovasc Nurs. (2007) 6:105–11. doi: 10.1016/j.ejcnurse.2006.05.005, PMID: 16839819

[23] Bjorshol CA Lindner TW Soreide E Moen L Sunde K. Hospital employees improve basic life support skills and confidence with a personal resuscitation manikin and a 24-min video instruction. Resuscitation. (2009) 80:898–902. doi: 10.1016/j.resuscitation.2009.06.009, PMID: 19573973

[24] Meissner TM Kloppe C Hanefeld C. Basic life support skills of high school students before and after cardiopulmonary resuscitation training: a longitudinal investigation. Scand J Trauma Resusc Emerg Med. (2012) 20:31. doi: 10.1186/1757-7241-20-31, PMID: 22502917 PMC3353161

[25] Monteiro MLRBP Ferraz AIB Rodrigues FMP. Assessment of knowledge and self-efficacy before and after teaching basic life support to schoolchildren. Rev Paul Pediatr. (2021) 39:2019143. doi: 10.1590/1984-0462/2021/39/2019143 PMC7401502 32756758

[26] Ramesh AC Hariprasad KV Abhishek KB Murthy MRK Edison M Hoek TLV. Teaching hands-only CPR (HOCPR) skills to 8th-grade students in urban Bengaluru: development of a comprehensive hands-only CPR programme for high school students. Indian J Anaesth. (2022) 66:140–5. doi: 10.4103/ija.ija_685_21, PMID: 35359484 PMC8963233

[27] Del Rios M Han J Cano A Ramirez V Morales G Campbell TL. Pay it forward: high school video-based instruction can disseminate CPR knowledge in priority neighborhoods. West J Emerg Med. (2018) 19:423–9. doi: 10.5811/westjem.2017.10.35108, PMID: 29560076 PMC5851521

[28] Pleasant A Maish C O’Leary C Carmona RH. A theory-based self-report measure of health literacy: the Calgary charter on health literacy scale. Methodol Innov. (2018) 11:205979911881439. doi: 10.1177/2059799118814394

[29] Ahmed AAA Al-Shami AM Jamshed S Fata Nahas AR. Development of questionnaire on awareness and action towards symptoms and risk factors of heart attack and stroke among a Malaysian population. BMC Public Health. (2019) 19:1300. doi: 10.1186/s12889-019-7596-1, PMID: 31619202 PMC6796340

[30] Riegel B McKinley S Moser DK Meischke H Doering L Dracup K. Psychometric evaluation of the acute coronary syndrome (ACS) response index. Res Nurs Health. (2007) 30:584–94. doi: 10.1002/nur.20213, PMID: 18022812

[31] Tang HM Wu X Jin Y Jin YQ Wang ZJ Luo JY. Shorter training intervals increase high school students’ awareness of cardiopulmonary resuscitation: a questionnaire study. J Int Med Res. (2020) 48:300060519897692. doi: 10.1177/0300060519897692, PMID: 31948297 PMC7113696

[32] Daniel WW ed. Biostatistics: A foundation for analysis in the health sciences. 7th ed. New York: John Wiley & Sons (1999).

[33] Akel M Fahs I Salameh P Godeau E. Are Lebanese schools adopting a health promotion approach in their curricula? Health Educ J. (2018) 78:476–85. doi: 10.1177/0017896918801716

[34] Bouclaous C Daher R Osseily W Rosário R Hamam H. Association between health and levels of health literacy in 13-to 16-year-old adolescents during the COVID-19 pandemic: the case of Lebanon. Can J Sch Psychol. (2023) 38:302–16. doi: 10.1177/08295735231197344

[35] Bouclaous CH Alrazim A Chababi J Jamaleddine W Nassar E Maalouf A. Association between sources of sexuality education, sexual beliefs and behaviours in Lebanese young adults: a university-based cross-sectional study. Sex Educ. (2021) 21:1–12. doi: 10.1080/14681811.2020.1722624

[36] Mukattash TL Shara M Jarab AS Al-Azzam SI Almaaytah A Al Hamarneh YN. Public knowledge and awareness of cardiovascular disease and its risk factors: a cross-sectional study of 1000 Jordanians. Int J Pharm Pract. (2012) 20:367–76. doi: 10.1111/j.2042-7174.2012.00208.x, PMID: 23134095

[37] Awad A Al-Nafisi H. Public knowledge of cardiovascular disease and its risk factors in Kuwait: a cross-sectional survey. BMC Public Health. (2014) 14:1131. doi: 10.1186/1471-2458-14-1131, PMID: 25367768 PMC4237772

[38] Mata J Frank R Gigerenzer G. Symptom recognition of heart attack and stroke in nine European countries: a representative survey. Health Expect. (2014) 17:376–87. doi: 10.1111/j.1369-7625.2011.00764.x 22390229 PMC5060725

[39] Han CH Kim H Lee S Chung JH. Knowledge and poor understanding factors of stroke and heart attack symptoms. Int J Environ Res Public Health. (2019) 16:3665. doi: 10.3390/ijerph16193665, PMID: 31569534 PMC6801587

[40] Khan IA Karim HMR Panda CK Ahmed G Nayak S. Atypical presentations of myocardial infarction: a systematic review of case reports. Cureus. (2023) 15:35492. doi: 10.7759/cureus.35492 PMC10048062 36999116

[41] Connolly M Toner P Connolly D McCluskey DR. The “ABC for life” programme—teaching basic life support in schools. Resuscitation. (2007) 72:270–9. doi: 10.1016/j.resuscitation.2006.06.031, PMID: 17134814

[42] Hatzakis KD Kritsotakis EI Karadimitri S Sikioti T Androulaki ZD. Community cardiopulmonary resuscitation training in Greece. Res Nurs Health. (2008) 31:165–71. doi: 10.1002/nur.20244, PMID: 18183565

[43] Chen M Wang Y Li X Hou L Wang Y Liu J. Public knowledge and attitudes towards bystander cardiopulmonary resuscitation in China. Bio Med Res Int. (2017) 2017:1–7. doi: 10.1155/2017/3250485 PMC5359437 28367441

[44] Ojifinni K Motara F Laher AE. Knowledge, attitudes and perceptions regarding basic life support among teachers in training. Cureus. (2019) 11:6302. doi: 10.7759/cureus.6302 PMC6944153 31938594

[45] Alwidyan M Alkhatib Z Alrawashdeh A Oteir A Khasawneh E Alqudah Z. Knowledge and willingness of schoolteachers in Jordan to perform CPR: a cross-sectional study. BMJ Open. (2023) 13:e073080. doi: 10.1136/bmjopen-2023-073080, PMID: 37553198 PMC10414105

[46] Vaillancourt C Kasaboski A Charette M Islam R Osmond M Wells GA. Barriers and facilitators to CPR training and performing CPR in an older population most likely to witness cardiac arrest: a national survey. Resuscitation. (2013) 84:1747–52. doi: 10.1016/j.resuscitation.2013.08.001, PMID: 23989115

[47] Farquharson B Dixon D Williams B. The psychological and behavioural factors associated with laypeople initiating CPR for out-of-hospital cardiac arrest: a systematic review. BMC Cardiovasc Disord [Internet]. (2023) 23:19. doi: 10.1186/s12872-022-02904-2, PMID: 36639764 PMC9840280

[48] W.H.O. UNESCO. Making every school a health-promoting school: Global standards and indicators for health-promoting schools and systems. WHO UNESCO [internet]. (2021); Available at: https://www.who.int/publications/i/item/9789240025059

[49] Bouclaous C Salem S Ghanem A Saade N El Haddad J Bou Malham M. Health literacy levels and predictors among Lebanese adults visiting outpatient clinics in Beirut. Health Lit Res Pract. (2021) 5:e295–309. doi: 10.3928/24748307-20211012-02 34756119 PMC8579750

[50] Bouclaous C Azar LJ Barmo N Daher R Tabaja J El Hout G. Levels and correlates of numeracy skills in Lebanese adults with diabetes: a cross-sectional study. Int J Environ Res Public Health. (2022) 19:10557. doi: 10.3390/ijerph191710557, PMID: 36078271 PMC9517913

[51] Bouclaous C Al Kamand A Daher R Al Razim A Kaedbey HD. Digital health literacy and online information-seeking behavior of Lebanese university students in the time of the COVID-19 pandemic and infodemic. Nord J Digit Lit. (2023) 18:60–77. doi: 10.18261/njdl.18.1.6

[52] Blakeman JR Prasun MA Kim M. Predictors of acute coronary syndrome symptom knowledge, attitudes, and beliefs in adults without self-reported heart disease. Heart Lung. (2023) 60:102–7. doi: 10.1016/j.hrtlng.2023.03.006 36947932

[53] Einspruch EL Lynch B Aufderheide TP Nichol G Becker L. Retention of CPR skills learned in a traditional AHA Heartsaver course versus 30-min video self-training: a controlled randomized study. Resuscitation. (2007) 74:476–86. doi: 10.1016/j.resuscitation.2007.01.030, PMID: 17442479

[54] Hamasu S Morimoto T Kuramoto N Horiguchi M Iwami T Nishiyama C. Effects of BLS training on factors associated with attitude toward CPR in college students. Resuscitation. (2009) 80:359–64. doi: 10.1016/j.resuscitation.2008.11.023, PMID: 19181430

[55] Blewer AL Putt ME Becker LB Riegel BJ Li J Leary M. Video-only cardiopulmonary resuscitation education for high-risk families before hospital discharge: a multicenter pragmatic trial. Circ Cardiovasc Qual Outcomes. (2016) 9:740–8. doi: 10.1161/CIRCOUTCOMES.116.002493, PMID: 27703033 PMC5339028

[56] Xu Y Li J Wu Y Yue P Wu F Xu Y. An audio-visual review model enhanced one-year retention of cardiopulmonary resuscitation skills and knowledge: a randomized controlled trial. Int J Nurs Stud. (2020) 102:103451. doi: 10.1016/j.ijnurstu.2019.103451, PMID: 31734218

[57] Anderson L Brown JP Clark AM Dalal H Rossau HK Bridges C. Patient education in the management of coronary heart disease. Cochrane Database Syst Rev. (2017) 6:CD008895. doi: 10.1002/14651858.CD008895.pub3, PMID: 28658719 PMC6481392

[58] Fathi B Allahverdipour H Shaghaghi A Kousha A Jannati A. Challenges in developing health promoting schools’ project: application of global traits in local realm. Health Promot Perspect. (2014) 4:9–17. doi: 10.5681/hpp.2014.002 25097832 PMC4122035

[59] Bouclaous C Fadlallah N El Helou MO Dadaczynski K. University students’ experience of the Beirut port explosion: associations with subjective well-being and subjective symptoms of mental strain. J Ment Health. (2023) 32:602–11. doi: 10.1080/09638237.2022.2140785, PMID: 36322513

[60] Issa E El Choueiry F Alhajj M Shbaklo K Bouclaous C. A qualitative investigation of university students’ experience of the Beirut port explosion. J Loss Trauma. (2024) 29:454–473. doi: 10.1080/15325024.2023.2264785

